# Zika Virus, French Polynesia, South Pacific, 2013

**DOI:** 10.3201/eid2011.141380

**Published:** 2014-11

**Authors:** Van-Mai Cao-Lormeau

**Affiliations:** Institut Louis Malardé, Papeete, Tahiti, French Polynesia

**Keywords:** Zika virus, ZIKV, viruses, outbreak, dengue, dengue virus, Yap, Micronesia, French Polynesia, South Pacific

**In Response:** I want to respond to the letter by Hancock et al. (*1*) regarding the previously published letter, Zika Virus, French Polynesia, South Pacific, 2013 (*2*). My comment aims to clarify an inaccuracy in the following sentence. “In 2007, the first Zika outbreak ever reported outside Africa and Asia was retrospectively documented from biological samples of patients on Yap Island, Federated States of Micronesia, North Pacific, who had received an incorrect diagnosis of dengue virus (DENV)” (*2*).

I recognize that this sentence does not provide an accurate description of the efforts in Yap State to investigate the outbreak and further confirm that it was caused by Zika virus (ZIKV). As specified in the article by Lanciotti et al. (*3*), outbreak investigations continued although initial laboratory testing suggested dengue virus as the causative agent: “In April 2007, an epidemic of rash, conjunctivitis, and arthralgia was noted by physicians in Yap State, Federated States of Micronesia. Laboratory testing with a rapid assay suggested that a dengue virus (DENV) was the causative agent. In June 2007, samples were sent for confirmatory testing to the Arbovirus Diagnostic Laboratory at the Centers for Disease Control and Prevention (CDC, Fort Collins, CO, USA).” 

I apologize to the Yap Epinet Team for this inaccuracy, and I encourage the reader to consult the articles by Lanciotti et al. (*3*) and Duffy et al. (*4*) to get a complete description of the clinical and laboratory investigations conducted during the ZIKV outbreak in Yap State. If data and laboratory protocols (reverse transcription PCR) related to this first ZIKV outbreak in the Pacific had not been available to the scientific community, identification of ZIKV as the cause of an outbreak in French Polynesia in 2013 would have been greatly delayed.
